# Research and Development of a DNDC Online Model for Farmland Carbon Sequestration and GHG Emissions Mitigation in China

**DOI:** 10.3390/ijerph14121493

**Published:** 2017-12-01

**Authors:** Zaidi Jiang, Shan Yin, Xianxian Zhang, Changsheng Li, Guangrong Shen, Pei Zhou, Chunjiang Liu

**Affiliations:** 1 School of Agriculture and Biology and Research Centre for Low Carbon Agriculture, Shanghai Jiao Tong University, 800 Dongchuan Rd., Shanghai 200240, China; jzd930303@sjtu.edu.cn (Z.J.); xixizi01090@163.com (X.Z.); changsheng.li@unh.edu (C.L.); sgrong@sjtu.edu.cn (G.S.); zhoupei@sjtu.edu.cn (P.Z.); 2 Shanghai Urban Forest Research Station, State Forestry Administration, 800 Dongchuan Rd., Shanghai 200240, China; 3 Key Laboratory for Urban Agriculture, Chinese Ministry of Agriculture, 800 Dongchuan Rd., Shanghai 200240, China; 4 Eco-Environmental Protection Research Institute, Shanghai Academy of Agricultural Sciences, 1000 Jinqi Road, Shanghai 201403, China

**Keywords:** DNDC online model, agricultural managements, carbon sequestration, GHG emissions mitigation

## Abstract

Appropriate agricultural practices for carbon sequestration and emission mitigation have a significant influence on global climate change. However, various agricultural practices on farmland carbon sequestration usually have a major impact on greenhouse gas (GHG) emissions. It is very important to accurately quantify the effect of agricultural practices. This study developed a platform—the Denitrification Decomposition (DNDC) online model—for simulating and evaluating the agricultural carbon sequestration and emission mitigation based on the scientific process of the DNDC model, which is widely used in the simulation of soil carbon and nitrogen dynamics. After testing the adaptability of the platform on two sampling fields, it turned out that the simulated values matched the measured values well for crop yields and GHG emissions. We used the platform to estimate the effect of three carbon sequestration practices in a sampling field: nitrogen fertilization reduction, straw residue and midseason drainage. The results indicated the following: (1) moderate decrement of the nitrogen fertilization in the sampling field was able to decrease the N_2_O emission while maintaining the paddy rice yield; (2) ground straw residue had almost no influence on paddy rice yield, but the CH_4_ emission and the surface SOC concentration increased along with the quantity of the straw residue; (3) compared to continuous flooding, midseason drainage would not decrease the paddy rice yield and could lead to a drop in CH_4_ emission. Thus, this study established the DNDC online model, which is able to serve as a reference and support for the study and evaluation of the effects of agricultural practices on agricultural carbon sequestration and GHG emissions mitigation in China.

## 1. Introduction

Greenhouse gas (GHG) emissions from the farmland system have a significant impact on global climate change. Global agricultural activities contribute about 14% to the total GWP (Global Warming Potential) according to the report published by the Intergovernmental Panel on Climate Change (IPCC) [1]. The GHG emissions from agricultural production activities are mainly CH_4_ (25%), N_2_O (52%) and CO_2_ (23%) [2].

The agroecosystem is a relatively complex ecosystem. Crop production and GHG emissions are regulated by climate, soil and farming practices [3]. Therefore, application of appropriate carbon sequestration practices is profoundly significant for increasing crop production and mitigating GHG emissions [4]. Tillage, straw residue, nitrogen fertilization reduction and water management are common carbon sequestration practices [5]. Each of these agricultural practices has a large and varied impact on the GHG emissions. For example, straw returning could reduce CO_2_ emission by avoiding crop residue burning; however, it provides an extra carbon source for soil methanogenic bacteria, which would cause an increase in CH_4_ emissions [6]. Sufficient nitrogen fertilization not only guarantees crop yield directly but also promotes microbial activities on fixing soil carbon and emitting nitrogen oxides such as N_2_O [7]. Therefore, comprehensive evaluation of the benefits of each carbon sequestration practice is very important to formulate appropriate agricultural management strategies under different conditions of climate, soil and planting pattern.

Because of the spatial and temporal heterogeneity of GHG emissions existing in farmland soil, it is very difficult to conduct in situ measurements across different regional and time scales. In addition, it is quite inaccurate to estimate regional GHG emissions in the long term based on the data of a local short-term experiment. Thus, after comprehensive consideration of various influence factors of GHG emissions, many mathematical models, including emission inventories, empirical models and process models, are widely developed and applied in estimating GHG emissions [8]. Emission inventories or empirical models, recommended by IPCC, use emission coefficients related to various agricultural activities. However, the universality of empirical models deviates with the increase of the number of coefficients, and the uncertainty of the simulation results increases as well. However, process models, which parse the carbon and nitrogen transformation process, soil physical and chemical properties, climatic changes and plant growth into advanced physical, chemical and biological formulas, relatively accurately and reliably estimate the GHG emissions in agroecosystem. Process models, mainly including DayCent, APSIM, DNDC, WNMM, AgMod, etc., are based on biogeochemistry as a core mechanism [9].

Among these, the DNDC model is a computer model that contains a relatively large suite of biophysical and biogeochemical processes to describe complex transport and transformations of carbon and nitrogen in terrestrial ecosystems under both aerobic and anaerobic conditions [10]. It connects the chemical reactions in soil with the ecological drivers (such as atmosphere, soil, crops and farming practices) and environmental factors (such as temperature, moisture, pH, Eh, radiation) and simulates carbon and nitrogen dynamics in the complex systems to estimate and predict soil organic carbon (SOC) change, crop yield and GHG emissions [11]. The DNDC model was first published in 1992 by Professor Li Changsheng. At the international senior symposium, called “Evaluation of Soil Organic Matter Models Using Existing Long-Term Datasets” in Rothamsted, 1995, the DNDC model was evaluated as one of the best four models to be tested with 12 long-term datasets from nine different countries [12]. In 2002, Smith et al. tested the ability to estimate N_2_O emissions from agricultural soils of the DNDC model with measured data from two experimental sites in Canada. He reported that the DNDC model was more accurate than IPCC methodology at estimating N_2_O emissions at both sites [13]. Babu et al. conducted a field validation experiment of the DNDC model for CH_4_ and NO_x_ emissions from rice-based production systems of India in 2006 and found that the DNDC model satisfactorily simulated the seasonal variations in greenhouse gas emission from paddy fields with different land management [14]. In 2011, the result of the experiment conducted by Abdalla et al. indicated that the DNDC model could reliably estimate soil respiration from adjacent pasture and arable fields in the Irish midlands [15]. The result of the experiment conducted by Zhao et al. in 2016 indicated that the DNDC model accurately simulated rice yields and N loss from paddy fields under different fertilization methods [16]. After two decades of development, the DNDC model has been well validated and applied in more than 30 countries over the world.

Although the DNDC model is outstanding in terms of simulation accuracy, it has some shortcomings in terms of practicality: (1) The DNDC model requires corresponding parameters of climate, soil and agricultural management to simulate carbon and nitrogen dynamics. The lack of initial parameters leads to inaccuracy in results. In addition, it is very difficult and tedious for model users to acquire those parameters. (2) With the progress of research and the development of the DNDC model, more operational parameters will be added into the model itself. Model users need to manually update the application program. (3) Most of the progress models have unique system environment requirements. For instance, the DNDC model has to run on the Win32 platform.

Because of the significance of quantifying agricultural practices’ efficiency for carbon sequestration and the limitation of the GHG emissions simulation model, this study intended to develop a suitable online platform, named the DNDC online model, for quantifying the mitigation of GHG emissions and evaluating carbon sequestration for China’s agroecosystem. The platform could help users to (1) query necessary parameters for model simulation online directly; (2) run the simulation model online and acquire visual simulation results; (3) estimate the carbon sequestration effects of various agricultural practices; (4) download original simulated data for further research.

## 2. Design 

### 2.1. Overall Design

**Figure 1:**
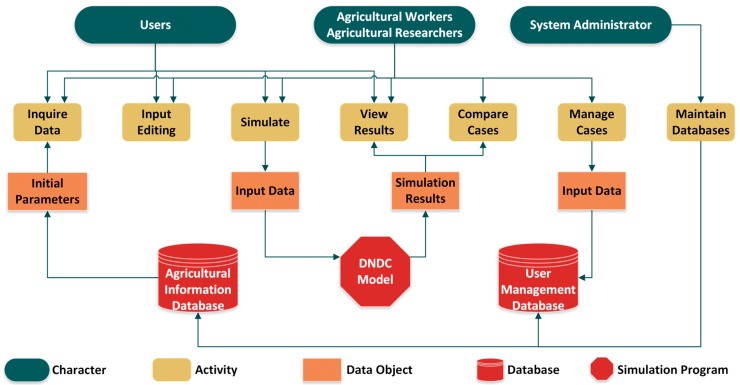
The logical structure diagram of the DNDC online model platform.

The DNDC online model used the original DNDC model’s scientific computation as its core mechanism. This study established the specification of the input/output process of the model program. In order to solve the difficulties of acquiring necessary parameters for the DNDC model, this study built an agricultural information database containing meteorological data, soil initial parameters, crop initial parameters and agricultural management information. Then, this study established an online web platform that was able to respond to users’ input operation, run the DNDC model simulation program and export visual diagrams of the results. Furthermore, this platform allows professional users like agricultural employees or agricultural researchers to save user-defined cases on the server and compare effects of different agricultural management. The agricultural information and user information were maintained by the system administrator. Thus, this study provided a convenient and flexible DNDC online model platform which was able to estimate the environmental physical and chemical properties as well as the GHG emissions mitigation due to different agricultural practices based on the simulation results of the DNDC model. The logical structure of the system is shown in Figure 1.

### 2.2. Detailed Design

According to characteristics of the original DNDC model and the objectives of this study, we analyzed and designed the database and the function modules of each part of the DNDC online model platform.

#### 2.2.1. Model Database

According to the initial parameter requirements of the DNDC model, this study built a geographical information system (GIS) database to provide reference information that users need to start model simulation. This database includes: (1) meteorology information such as daily temperature and precipitation; (2) soil properties such as SOC content, range of pH, bulk density, clay fraction and porosity; (3) crop properties such as biomass allocation, C/N ratio, water requirement and accumulated temperature; (4) local default agricultural management information such as the date of planting, tillage and harvest, the amount of fertilization and irrigation, etc.

The user management database in this platform (1) recorded the log of users’ login and registration information, (2) stored all user-defined cases, and meanwhile (3) responded to the add/delete/modify request of any case.

#### 2.2.2. Model Simulation Program

Because of the automatization and concurrency of the web platform, the original core process of the DNDC model program based on GDI+ and console I/O was not suitable for the online platform. The new model simulation program, named the China-DNDC model by the chief founder of the DNDC model—Professor Li Changsheng, was executed with shell I/O and transmitted data in the form of serialized data stream while sharing the same computation process as the original DNDC model. Users were able to run the model through the web application on the server. In addition, it is convenient for system administrators’ unified management of the model simulation program.

#### 2.2.3. Model User Interface

The web platform developed by this study was based on Browser/Server (B/S) mode, so the browser application on clients’ terminal serves as the front-end user interface. This platform provided front-page design and responding script to the user’s interface operation. The responding application on the server executed the model simulation program after it received the submitted request and returned simulation results in binary data. The user interface displayed the simulation results as tables and diagrams. The platform user interface with simple operation and elegant appearance provides convenient and flexible interaction between network users and DNDC online model simulation program.

## 3. Implementation

### 3.1. Construction of the Model Database

The database of DNDC online model contains two major parts. The agricultural information database includes all necessary initial parameters required by the original DNDC model. According to different characteristics of the information, this study designed three types of data structure.

Meteorological information was stored in text files according to the original DNDC model’s climate file standard, indexed by the meteorological station number of its geographical location, and containing the daily maximum temperature, minimum temperature and precipitation. Geographical information, soil default parameters and crop default parameters were stored in MySQL data tables. Geographical information contained the administrative region, latitude, longitude, SOC content, range of pH, bulk density and nitrogen content in precipitation, etc. of each county site. Soil default parameters contained the porosity, saturated conductivity, field capacity, specific heat, etc. of each land-use type and soil texture. Crop default parameters contained the biomass, distribution, C/N ratio, water requirement, optimum temperature, accumulated temperature, etc. of each type of crop. The agricultural management information was stored in JavaScript Object Notation (JSON) text indexed by the county site number. It contained the distinguishing information of agricultural management that is mostly applied in the county site.

The user management database included the user data table and the case data table. The former table contained the user identification code, identity authentication keys and contact information. The latter table contained the necessary input data for the model and the output data of the simulation results in the form of JSON text, related to the user data table by the user identification code.

### 3.2. Data Collection

The complexity of the DNDC model determined the difficulty of acquiring all the necessary information and parameters. For example, the simulation of a county site required 45 parameters of soil property, 26 parameters of crop property and 39 parameters of agricultural managements. This study obtained the GIS data of 2483 county sites and meteorology data of 610 stations collected by Professor Li during his research on greenhouse gas emissions from croplands in China [17]. Furthermore, this study was kindly granted the source code of DNDC95 program and all the predefined parameters of different types of crop and soil texture. The daily meteorology data were downloaded from the website of National Center for Atmospheric Research of the USA (NCAR). The nitrogen deposition data in atmosphere were calculated by five three-dimensional chemical models: GCTM, GRANTOUR, IMAGES, MOGUNTIA and ECHAM [18]. The soil texture, bulk density, SOC content and range of pH of each county site were collected from the second national soil survey, according to Chinese Soil Atlas [19]. The planting area of each type of crop in each county site were measured by remote sensing with reference to the ratio between each pair of crop types provided in China Agriculture Yearbook. The date of planting and harvesting of each type of crop was from Atlas of Agroclimatic Resources in China published by China Meteorological Press [20].

### 3.3. Compile of the Model Computation Process

Based on the original source code of DNDC95, while maintaining the core scientific computation process, this study designed a new model class. By overwriting the I/O functions, the new model simulation program was able to receive and send data in the form of serialized data stream, thusly avoiding file concurrency conflicts in multi-process tasks. This study also overwrote some interactive functions to support shell command and multithreading. Furthermore, this study designed a universal function template to handle different agricultural activity events to facilitate functional extension in the future.

**Figure 2:**
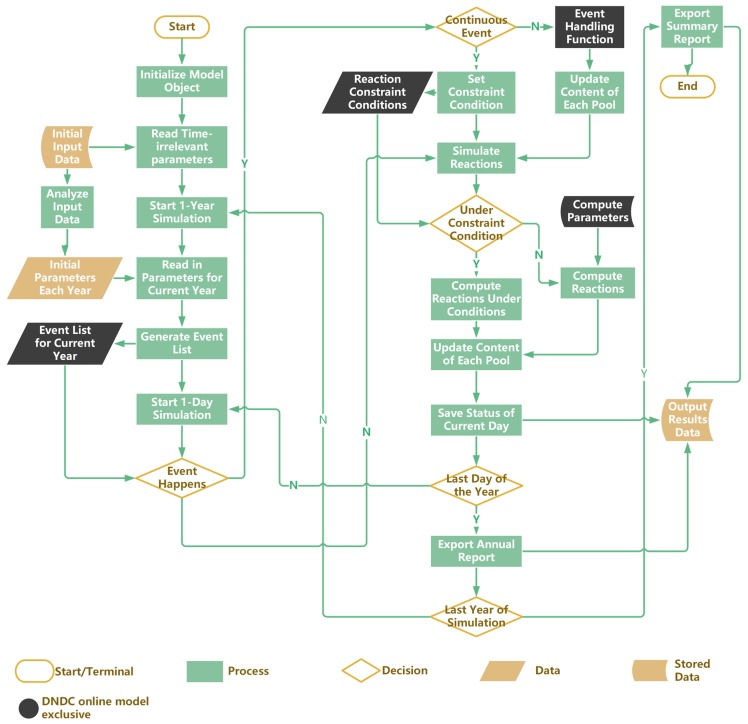
Abridged procedure flowchart of the DNDC online model simulation program.

We chose the open source programming language—Python—to establish the DNDC online model simulation program. Python has concise syntax and high extensibility, and it is very easy to read the code. It also has a rich library of scientific computing tools. On condition of preserving the core computation process, this study rewrote the constructor function, input functions and output functions. The data of farmland management events were stored in a linked matrix with a unique event handling function for each kind of management. Then we established two lists for event handling functions and constraint conditions in the convenience of adding handling functions for the new type of agricultural management event in the future, such as biomass charcoal and nitrification inhibitor. The abridged procedures of the DNDC online model simulation program is shown in Figure 2.

### 3.4. Establishment of the Model Server

**Figure 3:**
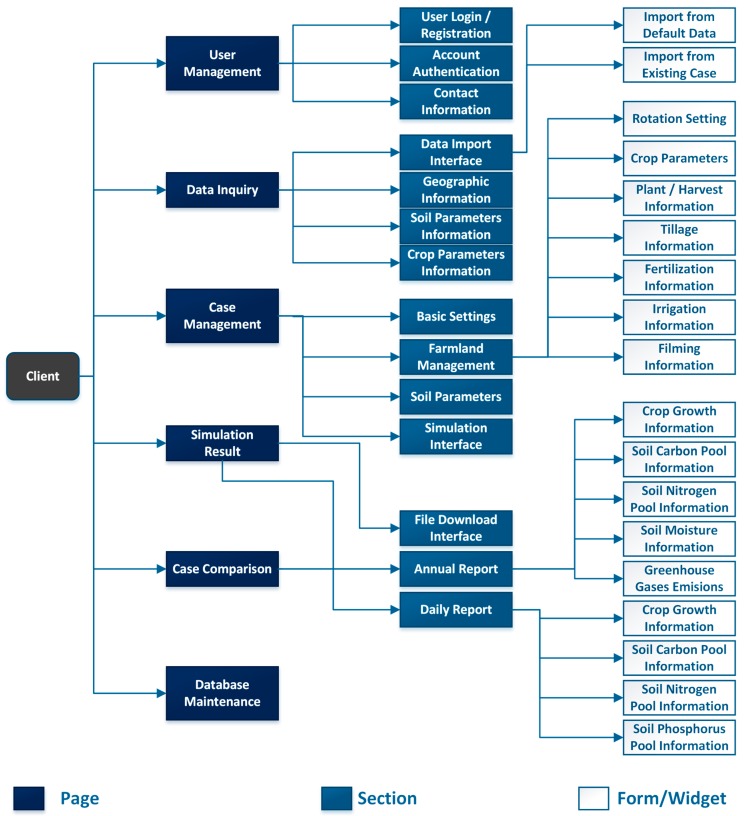
Structure of the DNDC online model’s user interface.

The frontend of the platform contained five major components: user account management page, user-defined case management page, default data inquiry page, model simulation page and case comparison page. Besides those, there were also the website index page, login and registration page and backstage maintenance page. First of all, the client-end user could make an inquiry for agricultural information from the server database, and import the acquired data input for a user-defined case. Next, the user could edit the input parameters in the case and submit them to the server. The server would receive submitted data and execute model programs to simulate the biogeochemical dynamics then return the simulation results data back to the client-end. The responding script in the browser would display the returned data in the form of tables and diagrams and allow user to download daily simulation report file. Additionally, a database maintenance page was also part of the platform’s interface, as shown in Figure 3.

**Figure 4:**
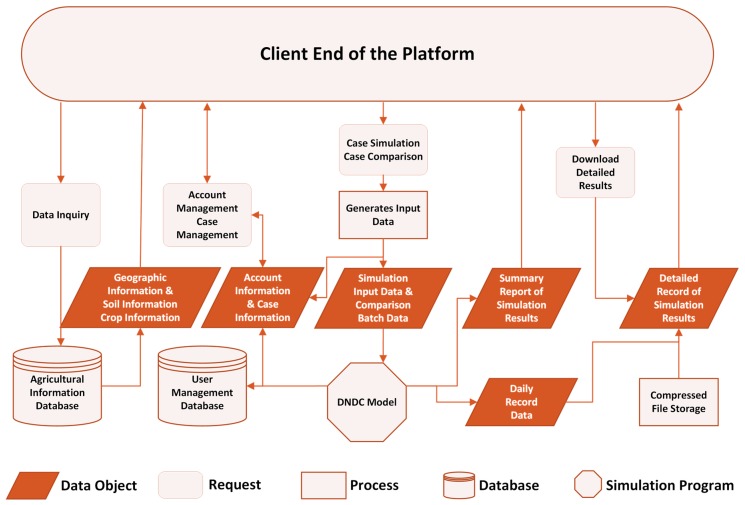
Procedure flowchart of the DNDC online model’s server.

The server for the DNDC online model was based on Linux + Apache + MySQL + PHP (LAMP) framework. This framework is popular among web developers due to its low cost, simplicity and stability [21]. The Client Interface of the platform was based on the W3C Recommendation of HTML5 and CSS3. This study compiled the browser-end script by means of the open source JavaScript library—jQuery and built the frame and components of the front pages with the adaptive tool set—jQuery UI. In the end, this study erected the database management tool—PHPMyAdmin—to help system administrators remotely maintain the MySQL database in the server. The procedure for the server’s application is shown in Figure 4.

## 4. Application

### 4.1. Validation of the DNDC Online Model

**Figure 5:**
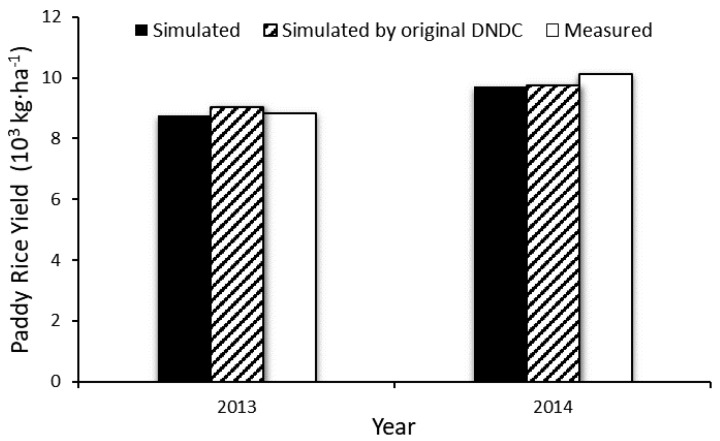
Comparison between simulated and measured values of crop yield [22].

**Figure 6:**
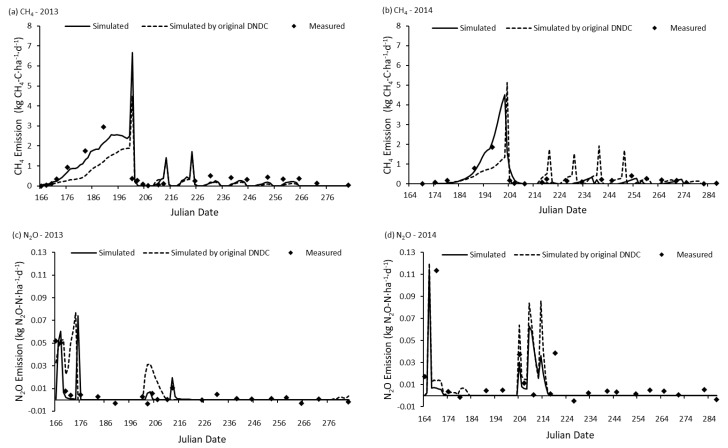
Comparison between simulated and measured value of daily CH_4_ and N_2_O emissions [22].

To validate the accuracy and applicability of DNDC online model, this study used it to simulate the paddy rice yield and CH_4_, N_2_O emission of the experimental field in Shanghai Engineering Research Center of Low-Carbon Agriculture at Zhuanghang experimental station (30°53′ N, 121°23′ E) which was published by Sun et al. (2016) [22]. The simulated results were compared with the in-situ observed data and the result simulated by the original DNDC model (ver.95) (Figure 5 and Figure 6). The comparison suggested that the DNDC online model well estimated the rice yield and CH_4_ and N_2_O emissions. In addition, it suggested that there was no significant difference between the results simulated by DNDC online model and those simulated by the original DNDC model.

**Figure 7:**
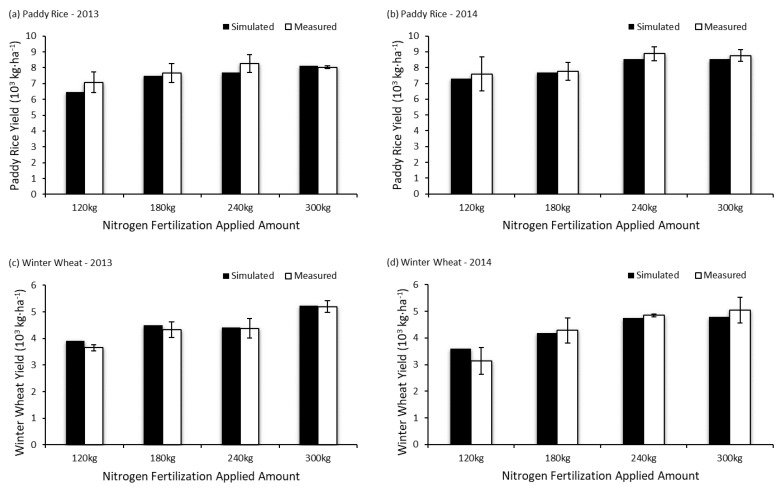
Comparison between simulated and measured crop yield in rice-wheat rotation system [23].

This study continued to simulate the crop yield of a rice-wheat rotation system with the local rice-wheat production pattern (300/225 kg N/ha fertilization applied during the rice/wheat cultivation period) applied in Changshu Agro-ecological Experimental Station (31°32′ N, 120°41′ E) of the Chinese Academy of Sciences in Jiangsu Province and compared the result with data measured by Xia et al. [23] (Table 1 and Figure 7). The result of two-factor ANOVA with replication showed that there was no significant difference (*p* > 0.05) between simulated and measured data in both paddy rice yield and winter wheat yield.

Thus, the DNDC online model was proven applicable in estimating crop yield and GHG emissions by the two cases above.

### 4.2. Prediction and Evaluation of the Carbon Sequestration Practice Effect

Utilizing the DNDC online model, this study estimated the effect of three common carbon sequestration measures: nitrogen fertilization reduction, straw residue and midseason drainage. According to the crop yield and CH_4_, N_2_O emissions in simulated results, this study evaluated the carbon sequestration practices and gave the recommendation.

#### 4.2.1. Nitrogen Fertilization Reduction

**Figure 8:**
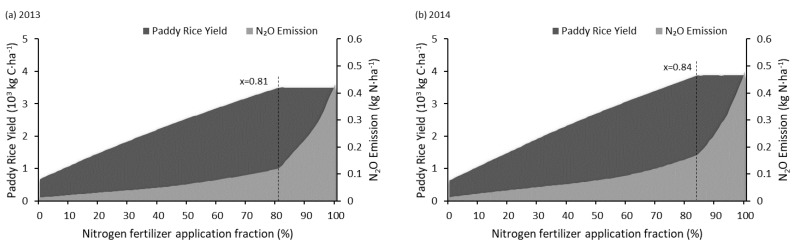
Changes in crop yield and N_2_O emission with different nitrogen fertilization fraction.

The actual nitrogen fertilization applied in the experimental field in Shanghai Engineering Research Center of Low-Carbon Agriculture at Zhuanghang experimental station is 225 kg N/ha at a ratio of 5:3:2 as base, tillering and heading fertilizers in the form of Urea. The results of the experiment conducted by Qiu et al. in 2009 showed that reducing the application rates of synthetic fertilizer could decrease N_2_O emissions while at the same time maintaining existing crop yields [24]. This study ran a model built-in Monte-Carlo analysis on nitrogen fertilization with the given ratio and the varying quantity between 0~100%. The analysis result (Figure 8) suggested that: crop yield had no significant variation with the change of fertilization applied quantity while it was above 84% of its original degree (189 kg N/ha). On the other hand, the annual N_2_O emission increased along with the fertilization application quantity. Thus, the DNDC online model reported a recommendation value of 84% (189 kg N/ha) for this case.

#### 4.2.2. Straw Residue

**Figure 9:**
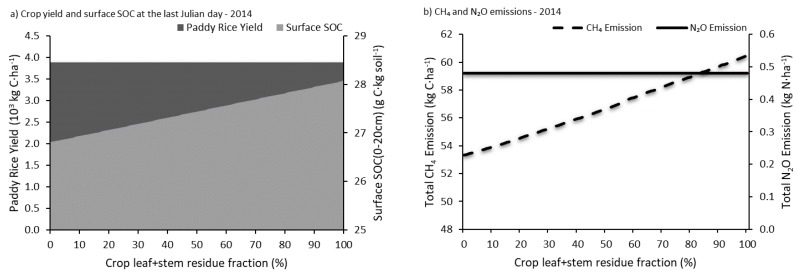
Changes in crop yield, CH_4_ emission, N_2_O emission and surface SOC at the last Julian day with different straw residue fraction.

In the experiment referred to above, almost all of the leaves and stems of the crop were harvested along with the grain. This study ran a Monte-Carlo analysis on ground straw residue with varying quantities between 0~100%. The analysis result (Figure 9) suggests that crop yield and N_2_O emission had no significant variation with the change of straw residue quantity. However, the CH_4_ emission and the surface SOC (0~20 cm) increased along with the straw residue fraction, which means straw residue had a negative effect on GHG emission mitigation but a positive effect on soil carbon sequestration, in agreement with the conclusion reached by Qiu et al. in 2009 [24].

#### 4.2.3. Midseason Drainage

**Figure 10:**
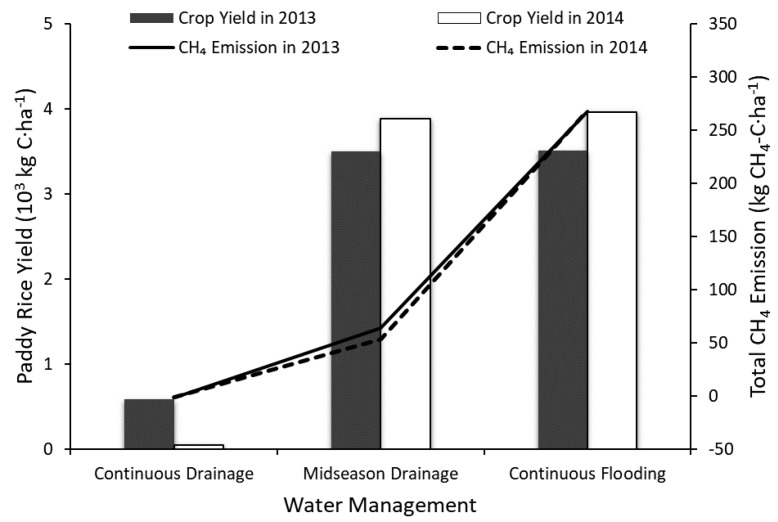
Changes in crop yield and CH_4_ emission with different field irrigation management.

In the experiment referred to above, the crop experienced alternative wet and dry flooding from 29 July to 10 October 2013 and from 4 August to 8 October 2014. This study simulated two other cases with different situations: continuous flooding from the base fertilization (13 June 2013 and 14 June 2014) to the end of the heading stage (10 October 2013 and 8 October 2014) and continuous drainage through the growing season. According to the simulated results of crop yield and CH_4_ emission (Figure 10), there is almost no difference in crop yield between the case applying midseason drainage and the case applying continuous flooding. The case applying continuous drainage showed a great drop in crop yield (83.28~98.71%) but exhibited a very slight amount of CH_4_ emission. The CH_4_ emission in the case applying midseason drainage was about 19.87~23.86% of that in the case applying continuous flooding. The result suggested that midseason drainage was very effective in CH_4_ emission mitigation while maintaining the paddy rice yield in the experimental field, which accords with the conclusion by Li et al. in 2002 [25].

All these three cases above indicated that the DNDC online model was an efficient tool to estimate the carbon and nitrogen cycling in agricultural ecosystems, such as soil carbon sequestration, crop yield and GHG emissions.

## 5. Conclusions

Given that global warming is becoming an increasingly severe problem, this study developed the DNDC online model for estimating GHG emissions and evaluating the effect of carbon sequestration practices based on the universal biogeochemical model—the DNDC model—by means of computer network and database technology. This study established a database which contains nation-wide agricultural information in China and developed an optimized model simulation program. During the program optimization, this study introduced new operational functions, designed an extensive interface for convenience of function extension and accuracy improvement, which has profound significance in the consummation and promotion of the DNDC model. The DNDC online model is proved effective in estimating the crop yield and GHG emissions as it was validated with the in-situ observed data and result simulated by the original DNDC (ver.95) in two sample sites. In addition, it is also proved efficient in evaluating the effect of different agricultural practices on carbon sequestration and GHG emissions mitigation as this study utilized the DNDC online model to estimate the effect of three common agricultural practices—nitrogen fertilization reduction, straw residue and midseason drainage. Thus, this study provided a useful tool for the research of agroecosystem carbon sequestration and GHG emissions mitigation in China.

## Figures and Tables

**Table 1:** Simulated and measured yield of paddy rice and winter wheat in the 2013 and 2014 growing seasons [23].

Nitrogen Fertilization (kg/ha)	Paddy Rice Yield (kg)				Winter Wheat Yield (kg)			
	2013		2014		2013		2014	
	Measured	Simulated	Measured	Simulated	Measured	Simulated	Measured	Simulated
120	7079 ± 645	6460	7598 ± 1077	7292	3649 ± 121	3913	3138 ± 512	3593
180	7655 ± 601	7472	7768 ± 570	7705	4329 ± 296	4500	4281 ± 465	4186
240	8273 ± 569	7705	8880 ± 435	8525	4381 ± 370	4411	4849 ± 56	4748
300	8030 ± 101	8115	8761 ± 369	8545	5200 ± 220	5235	5041 ± 481	4802
